# Systemic Lupus Erythematosus and DNA Degradation and Elimination Defects

**DOI:** 10.3389/fimmu.2019.01697

**Published:** 2019-08-07

**Authors:** Borros Arneth

**Affiliations:** Institute of Laboratory Medicine and Pathobiochemistry, Molecular Diagnostics, University Hospital of Giessen and Marburg, Justus Liebig University Giessen, Giessen, Germany

**Keywords:** SLE, DNA-degradation, DNA-elimination, SLE-Systemic Lupus Nephritis, DNA-anti DNA

## Abstract

**Introduction:** Systemic lupus erythematosus (SLE) is a chronic autoimmune disease that is characterized by the production of autoantibodies specific for components of the cell nucleus and that causes damage to body tissues and organs. The pathogenesis of SLE remains unclear, with numerous studies pointing to a combination of genetic and environmental factors. A critical stage in SLE development is cell necrosis, in which undegraded chromatin and nucleoproteins are released into the blood, resulting in circulating cell-free DNA and serum nucleoproteins that trigger anti-dsDNA autoantibody production. This systematic literature review aimed to examine whether SLE stems from a DNA degradation and elimination defect.

**Materials and Methods:** An advanced literature search was conducted in PubMed using the following keywords: [(“SLE” OR “Systemic Lupus Erythematosus” OR “Lupus”)] AND [(“DNA” OR “DNA Degradation”)] AND [(“Defect Elimination”)]. More articles were obtained from the references of the identified articles and basic Google searches. Twenty-five peer-reviewed articles published within the past 10 years (2007–2018) were included for review.

**Results:** The findings of each study are summarized in **Tables 1**, **2**.

**Discussion and Conclusion:** The etiopathogenesis of SLE remains controversial, which limits therapeutic inventions for this disease. However, SLE is a DNA degradation and elimination disorder caused by uncleared histones and nuclear material that leak into the extracellular space and form cell-free DNA, triggering an immune response that destroys tissues and organs. Under normal conditions, apoptosis allows DNA and other nuclear material to be efficiently cleared through degradation and additional complex mechanisms such that this material does not trigger the immune system to produce nuclear autoantibodies.

## Introduction

Systemic lupus erythematosus (SLE) is an autoimmune disorder characterized by the production of antinuclear antibodies (ANAs) specific for nuclear antigens originating from uncleared apoptotic cells. The disease affects several organs of the body. Globally, ~5 million people suffer from SLE, which has no cure to date (1). Evidence from previous studies shows that environmentally induced defects and genetic abnormalities in immune cells, mutations in regulatory components involved in cellular apoptosis and defects in mechanisms of cellular debris clearance are key contributors to SLE pathogenesis (2). More precise evidence from genome-wide studies shows that SLE patients have mutations in the three prime repair exonuclease 1 (TREX1), autophagy-related 5 (ATG5), RAD518, and deoxyribonuclease I (DNASE1) genes, which are involved in the degradation of DNA, cell apoptosis and the elimination of cellular debris (3).

Abnormalities in cell death processes have been implicated in the production of nuclear autoantigens, which result in a systemic autoimmune response if imperfectly cleared. This effect has been observed in SLE and many other chronic inflammatory diseases (4). These cell death processes include apoptosis, necrosis (primary and secondary), NETosis (a neutrophil-specific type of cell death), autophagy, necroptosis, and pyroptosis (5). Through a complex series of events, the production of autoantigens stimulates the immune system to produce autoantibodies that combine with uncleared cellular debris in the blood or tissues, leading to the formation of immune complexes (ICs). When dendritic cells, macrophages, and phagocytes clear ICs from the blood, proinflammatory cytokines are secreted, which subsequently leads to inflammation (4). The continued production of antibodies due to uncleared tissue debris in the blood and tissues results in perpetual inflammation and tissue damage, a characteristic of SLE (4).

Mitochondria hyperpolarization, ROS production, lysosomal membrane disintegration, organelle and cellular swelling, and plasma membrane rupture characterize necrotic cell death, which is often regarded as inflammatory since the loss of plasma membrane integrity stimulates the release of autoantigens and damage-associated molecular patterns (DAMPs), which act as chemoattractants for inflammatory cells (5).

Conversely, deoxyribonucleic acid (DNA) is an essential molecule for life; it carries hereditary information and is necessary for all organisms (6). Various events can trigger the degradation of DNA into nucleotides by DNase. Apoptosis is a classic example of such an event. This process has been well-known for years, but recent developments show that improper DNA degradation can result in various diseases, such as autoinflammation, cancer, and cataracts (7). SLE is characterized by the production of ANAs, which are autoantibodies specific for a variety of nuclear autoantigens. Anti-dsDNA autoantibodies are among the ANAs used to diagnose SLE (8). Normally, dsDNA is present in the nucleus and mitochondria, where it is shielded from recognition by the immune system. DNase I destroys DNA in the extracellular space, whereas DNAse II and III degrade endosomal and cytoplasmic DNA. Defective cell apoptosis, NETosis, and elimination processes result in elevated levels of cell-free dsDNA (cfDNA) in SLE patients (8). Therefore, understanding the complex pathways and processes involved in the formation of autoantigens and autoantibodies can help in the diagnosis and treatment of various diseases, such as cancer and SLE. Furthermore, these defects can be prevented effectively if proper DNA degradation is facilitated through extrinsic factors, such as drugs. In this paper, we review the connection between SLE and defective DNA degradation.

## Materials and Methods

An advanced literature search was conducted to retrieve the most relevant articles from the evidence base. Additional methods of identifying relevant literature were also employed, including looking up sources in the reference lists of other research reports, manual searching of relevant journals, and searching by author.

The advanced search was done using PubMed only because PubMed features top resources in chemicals and bioassays, DNA and RNA, and genes and expression, among other topics. Boolean connectors (“AND” and “OR”) were used to connect keywords in the search boxes of PubMed. The following search terms were used: [(“SLE” OR “Systemic Lupus Erythematosus” OR “Lupus”)] AND [(“DNA” OR “DNA Degradation”)] AND [(“Defect Elimination”)]. When the search terms were applied, 2,728 results were retrieved, including 1,047 articles published within the last 10 years. Only articles with free full texts were selected (490 articles). After screening the relevance of these articles using the titles, 42 articles were selected. The selected articles were then emailed to the researcher directly from PubMed, and their abstracts were further screened for relevance; 23 articles were retrieved and included in this review.

However, before implementing the advanced search strategy, a basic Google search was conducted as a prescreen search, which yielded 144,000 results when the following keywords were used: “Systemic Lupus Erythematosus,” “SLE,” “DNA degradation,” and “Defect elimination.” Four articles were retrieved, of which two were excluded; one of the excluded articles discussed how circulating free DNA may signal SLE severity and be used to monitor therapy (9), and the other article was excluded because it was outdated despite being relevant (10). Regarding the articles that were retrieved and included, one was about the relationship between the presence of SLE and the failure of DNA degradation (11), whereas the other was about the important role of neutrophil extracellular traps (NETs) in SLE (12). Therefore, 25 articles were included in this review.

## Results

**Figure 1:**
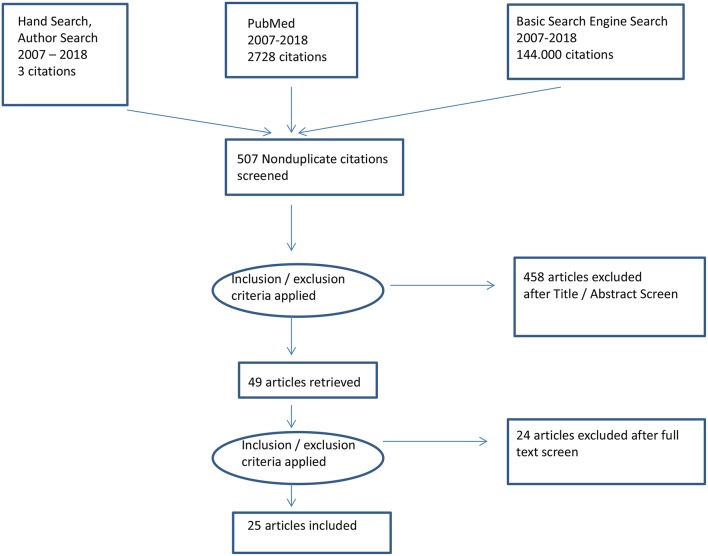
PRISMA diagram of the search strategy.

Twenty-five articles were retrieved for review by using the above search strategy (see Table 1). Figure 1 shows a PRISMA diagram of the search strategy described above.

**Table 1:** Retrieved articles selected for review.

**References**	**Year of publication**
Hendy et al. (9)	2016
Leffler (11)	2015
Wang et al. (12)	2015
Zhang et al. (13)	2014
Garcia-Romo et al. (14)	2011
Brightbill et al. (15)	2017
Niu et al. (16)	2017
Balada et al. (17)	2017
Nawrocki et al. (18)	2017
Yang et al. (19)	2015
Huang et al. (20)	2016
Gupta et al. (21)	2016
Hong et al. (22)	2017
Shu et al. (23)	2017
Zhu et al. (24)	2016
Sisirak et al. (25)	2016
Panza et al. (26)	2016
Yeung et al. (27)	2017
Steri et al. (28)	2017
Reddy et al. (29)	2017
Virdis et al. (30)	2015
Ikeda et al. (31)	2017
Li et al. (32)	2017
Sakai et al. (33)	2017
Stearns et al. (34)	2016

### Findings

This subsection will summarize the findings of each study; the findings will then be coded and categorized in the Discussion section. Tables 1, 2 summarize the retrieved studies considered in the discussion.

**Table 2:** Findings from each study.

**References**	**Findings**
Hendy et al. (9)	SLE patients have elevated levels of serum circulating cfDNA compared to healthy individuals. The elevated levels of serum dsDNA in SLE patients correlate with disease activity (9).
Leffler et al. (11)	Serum from SLE patients exhibits a very limited ability to degrade DNA from NETs and primary and secondary necrotic cells (11).
Wang H et al. (12)	mtDNA was detected in NETs, and elevated levels of anti-mtDNA antibodies were detected in serum from SLE patients. High levels of mtDNA in NETs and serum anti-mtDNA antibodies correlate with PDCs and type 1 IFN-α (12).
Zhang et al. (13)	SLE patients have very high serum levels of circulating cfDNA, which positively correlate with lupus nephritis (LN) activity. Further analysis showed that SLE patients with high levels of low-density granulocytes in peripheral blood mononuclear cells have high serum cfDNA levels and severe disease. This finding was attributed to lower DNase 1 activity in SLE patients than in healthy individuals (13).
Garcia-Romo et al. (14)	IFN-α stimulates neutrophils in SLE patients to form NETs in the presence of anti-ribonucleoprotein (RNP) antibodies. The formed NETs contain non-engulfed chromatin that facilitates DNA uptake by PDCs. NETs are the source of the elevated cfDNA levels in SLE patients (14).
Brightbill et al. (15)	Selective inhibition of NF-κB-inducing kinase (NIK) leads to improved survival of SLE mice *in vivo*. NIK mediates the activation of TNF proteins that have been implicated in SLE pathogenesis (15).
Niu et al. (16)	PBX1 has a direct regulatory effect on genes associated with T cell activation, and the PBX1-d isoform is associated with lupus. PBX1–d lacks a DNA-binding domain, and its expression in SLE patients leads to the production of autoreactive CD4+ T cells (16).
Balada et al. (17)	DUSP23 is overexpressed in SLE, which is linked to the expression of DNA methyltransferases (DNMTs) in CD4+ T cells (17).
Nawrocki et al. (18)	SLE is associated with decreased DNA methyltransferase mRNA expression levels (18).
Yang et al. (19)	Severe nephropathy in SLE can be indicated by IgG and concurrent reactivity to anti-dsDNA, anti-nucleosome, and anti-histone antibodies (19).
Huang et al. (20)	T cell vaccination helps improve and regulate the manifestations of SLE (20).
Gupta et al. (21)	Anti-IFN-γ autoantibodies are linked to aggressive SLE (21).
Hong et al. (22)	The hypermethylation of CD3Z is linked to severe clinical manifestations of SLE. CD3Z and VHL hypermethylation is associated with SLE. CD3Z hypermethylation is potentially an environmentally induced epigenetic modification (22).
Shu et al. (23)	Histone deacetylase inhibitors (HDACi), such as trichostatin (TSA), repress IRF5 and hence have therapeutic potential in patients with SLE. TSA-mediated inhibition of IRF5 binding to RNA polymerase II, HDAC3, DNA Sp1, and p300 in children with SLE suggests that SLE is associated with DNA degradation abnormalities and elimination defects (23).
Zhu et al. (24)	Several differentially expressed genes in SLE are involved in the IFN and TLR signaling pathways. The presence of hypomethylated upregulated genes and hypermethylated downregulated genes in SLE patients indicates that DNA methylation plays a role in SLE development (24).
Sisirak et al. (25)	The tolerance mechanism for SLE is marked by the digestion of chromatin in microparticles from dying cells. Mice and patients without the DNASE1L3 enzyme produce anti-dsDNA antibodies specific for serum dsDNA; hence, defective DNA degradation is associated with SLE (25).
Panza et al. (26)	PK201/CAT plasmid (PK) DNA and histone 4 (H4) assays are reliable for the differential diagnosis of SLE. Anti-PK/H4 antigens correlate with the level of SLE disease activity, suggesting a DNA damage defect in this disease (26).
Yeung et al. (27)	Unfractionated white blood cells can be used to indicate abnormal DNA methylation in SLE (27).
Steri et al. (28)	A variant of TNFSF13B is associated with SLE. Serum TNFSF13B correlates with serum dsDNA autoantigens in patients with SLE (28).
Reddy et al. (29)	Lower baseline serum IgM levels and sequential therapy with mycophenolate mofetil can predict IgM hypogammaglobulinemia after rituximab treatment in patients with SLE. No significant change in anti-dsDNA antibodies was observed in patients with initially higher levels, even after treatment. This finding suggests that high SLE disease activity is associated with dsDNA autoantigens in serum (29).
Virdis et al. (30)	Early treatment with hydroxychloroquine can curb the development of endothelial dysfunction in SLE patients (30).
Ikeda et al. (31)	The increase in anti-dsDNA antibodies in mice with higher SLE disease activity suggests that abnormal DNA degradation occurs in SLE (31).
Li et al. (32)	Aconitine can inhibit disease evolution and improve pathologic lesions in SLE. Elevated levels of dsDNA, ANAs, and proliferating cell nuclear antigen in mice with SLE before therapy show that SLE is characterized by defective DNA degradation and abnormal elimination of cellular debris. Aconitine therapy significantly reduced dsDNA autoantigens in serum and improved quality of life, indicating that defective DNA degradation is associated with SLE (32).
Sakai et al. (33)	OST inhibition can suppress autoantibody production in mice with SLE. Mutations in TREX1, such as D272fs, result in an inactive DNase in mice with SLE (33).
Stearns et al. (34)	The use of poly-L-lysine as a capture agent enhances the detection of SLE autoantibodies by ELISA. The detection of dsDNA, histones, RNP, SSA, and SSB antigens in serum from SLE patients using this assay showed that SLE is associated with abnormal DNA damage and elimination (34).

Several studies have been conducted in this field, with the most recent study reporting that NF-κB-inducing kinase (NIK) inhibition can improve outcomes in patients with SLE (35). Much remains unknown about SLE, and more studies are needed to explore more accurate and reliable ways of diagnosing and treating SLE. The following section of this paper will discuss these findings in the context of whether SLE is a DNA degradation defect.

## Discussion

### Defective Cell Death and cfDNA

Programmed cell death, termed apoptosis, is essential for homeostasis during both development and aging. Under normal conditions, apoptotic cells are engulfed into lysosomes within phagocytes and destroyed; this process occurs without inflammation (36). Many studies have maintained that SLE originates from the defective clearance of apoptotic remnants and NETs from the circulation and tissues (37–41). Thus, there is a consensus that SLE begins to develop when the clearance of dying cells is impaired. The clearance of cellular debris involves many ligands and receptors, and there must be sufficient bridging molecules between phagocytes and dying cells (42). This process is closely controlled and very intricate, but some studies have elucidated the details (43–45). Understanding this complex process has therapeutic potential, mainly in the timely prevention of SLE (45). Dying cells that are not quickly and efficiently removed go through secondary necrosis and subsequently burst, releasing nuclear material upon loss of an intact cell membrane (44).

The impaired clearance of apoptotic cells in SLE is due to their failed recognition by phagocytes because (a) the phagocytes are smaller (4), (b) the phagocytes have diminished and delayed phagocytic activity (46), (c) the phagocytes have reduced adherence (47), or (d) the differentiation of phagocytes from CD34-positive hematopoietic stem cells is reduced (48–50). Therefore, professional and non-professional phagocytes in patients with SLE have reduced phagocytic activity and are thus unable to clear NETs, and apoptosis remains incomplete (51).

During NETosis, NETs are generated by neutrophils upon pathogen stimulation to kill invading pathogens (40, 44). Cellular and nuclear components, including dsDNA, histones, neutrophil elastase, and myeloperoxidase, are released during NETosis. Proinflammatory components, including tumor necrosis factor (TNF)-α, interleukin (IL)-17, IL-8, and interferon (IFN)-γ, also trigger the production of NETs (13). Additionally, low-density granulocytes among peripheral blood mononuclear cells in SLE patients have been shown to form NETs (13). The formed NETs are normally degraded by DNase I, which hydrolyzes dsDNA through its endonuclease activity, thus breaking down chromatin during apoptosis (13).

Incomplete degradation of NETs by DNase I results in the accumulation of the remaining NETs, which react with other proteins to form complexes that are involved in autoimmune SLE (9, 12–14, 35). Elevated levels of undegraded residual NETs are the major source of serum dsDNA, also called circulating cfDNA, in SLE patients (51). The clearance of NETs entirely depends on an efficient and active DNase I enzyme. Defective DNase I leads to undegraded chromatin, which is positively related to SLE disease activity (9, 12, 51). The presence of mitochondrial DNA (mtDNA) in NETs and its positive correlation with plasmacytoid dendritic cells (PDCs) and type 1 IFN-α imply that SLE is a disorder of defective DNA clearance. Studies have shown that NETs in SLE patients activate PDCs and the IFN pathway, triggering the production of autoantigens (14). Therefore, defective DNA degradation leads to defective chromatin breakdown and is thus etiopathogenically involved in SLE development.

NIK usually engages non-canonical NF-κB to signal downstream of several TNF family members, including TWEAK, BAF, OX40, and CD40, which are involved in SLE pathogenesis (15). In addition to type I IFN and Toll-like receptor (TLR), numerous members of the TNF receptor superfamily (TNFRSF) are involved in SLE pathophysiology. CD40 and B cell-activating factor (BAFF) are essential for B cell survival and differentiation into autoantibody-generating plasma cells. CD40 ligand (CD40L) blockade showed promise in initial clinical trials of lupus, although therapeutic development was halted because of thrombotic side effects. In contrast, blockade of BAFF via belimumab is moderately effective and is currently the only new approved treatment for lupus (15).

### T Cell Activation and Defective DNA Degradation

Autoantigens that trigger the production of autoantibodies in SLE originate from cell death by apoptosis and NETosis. Dying cells usually undergo morphological changes, such as DNA fragmentation, shrinking, and blebbing of the plasma membrane; the nuclear autoantigens that are targeted in SLE are usually stored in these plasma membrane blebs (52). On the other hand, NETosis results in rupture of the plasma membrane, releasing the nuclear autoantigens that are targeted in SLE (53). These autoantigens resulting from apoptosis and NETosis trigger the production of antibodies that bind uncleared apoptotic material to form ICs. The mechanism through which these autoantibodies are produced is well-documented; T cells trigger B cells to differentiate, proliferate, and finally mature. Then, the T cells stimulate class switching of B cells to produce different classes of autoantibodies (16, 54). Due to alterations in T cells that modulate the production of autoantibodies specific for lupus autoantigens by B cells, lupus T cells have been categorized as abnormal (54).

**Figure 2:**
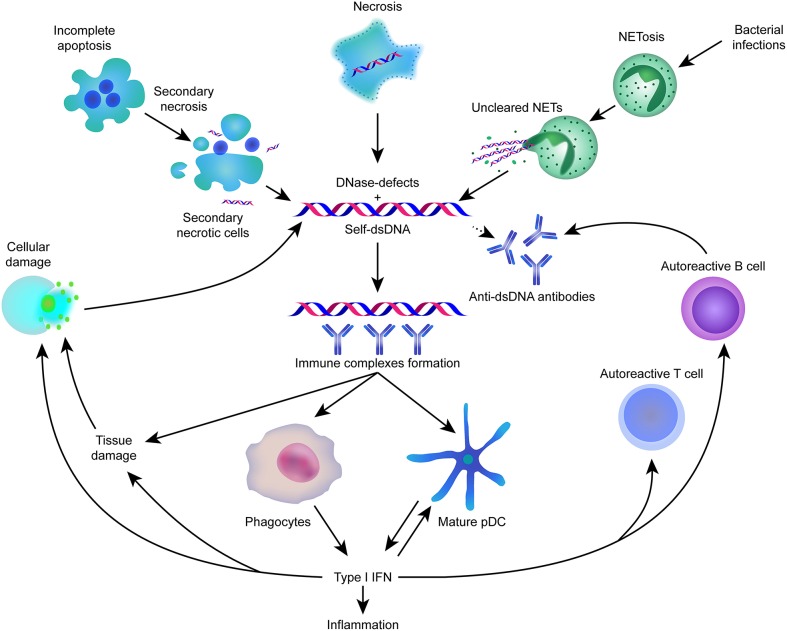
Self-dsDNA released during cell death plays an important role in the pathogenesis of SLE.

Lupus has also been shown to be associated with reduced expression of DNA cytosine-5-methyltransferases, which have hypomethylation activity that might be responsible for the production of autoreactive antibodies. Figure 2 gives an overview about the most important key-elements in the pathophysiology involved in systemic lupus erythematosus.

DNA methylation is essential for marking chromatin accessibility and regulating gene expression (55). DNA methylation involves the addition of a methyl group at the 5th carbon of cytosine residues in CG dinucleotides, and this process is usually involved in cell differentiation, X-chromosome inactivation, imprinting, and the suppression of transcriptional noise and parasitic DNA. Abnormalities in DNA methylation pathways have pathological consequences. For example, *de novo* mutation of the DNA methyltransferase DNMT3B leads to immunodeficiency-centromeric instability-facial anomalies syndrome (ICF syndrome). Complete DNMT1 deficiency is incompatible with life. Moreover, acquired defects in DNA methylation are related to diseases, including autoimmunity and cancer (55).

DNA methylation is principally a transcriptionally repressive epigenetic mark that renders chromatin inaccessible and promotes gene silencing through transcriptional repression; these events occur through different mechanisms, including the recruitment of methylcytosine binding domain-containing proteins that successively recruit histone deacetylases, which promote chromatin condensation (55).

Pbx1 is a member of the TALE family of homeodomain-containing transcription factors that regulates the DNA binding of Hox proteins. Pbx1 plays an essential role during organogenesis and development by integrating several signals via interactions with various partners, including Prep1 TALE proteins and Meis, that control chromatin remodeling and co-activator access. During immune system development, Pbx1 is vital for maintaining hematopoietic stem cell self-renewal and limiting myeloid maturation to preserve the differentiation ability of lymphoid progenitors. The absence of Pbx1 in embryonic stem cells leads to the failure to produce common lymphoid progenitors, the absence of NK and B cells, and the development of impaired T cells (16). In addition, Pbx1 regulates chromatin accessibility to various genes and is conserved between humans and mice. The Pbx1-d dominant-negative isoform is more commonly expressed in CD4^+^ T cells from lupus patients than in those from healthy controls. Pbx1-d is related to autoreactive T cell production in mice with the Sle1a1 lupus vulnerability locus (56).

Dual-specificity protein phosphatase 23 (DUSP23) activates the IFN and IL pathways via dephosphorylation (17), and these pathways are responsible for the formation of NETs, which are sources of cfDNA (17, 18, 57). DUSP3, DUSP22, and VH1 participate in both the IL and IFN signaling pathways mainly by dephosphorylating signal transducer and activator of transcription (STAT) proteins (37, 38). High IFN-α activity is frequently detected in sera from SLE patients (39). Moreover, patients with SLE exhibit a particular mRNA expression profile of IFN-dependent genes in leucocytes known as the IFN signature (17, 40, 41).

The presence of hypomethylated DNA in SLE patients implies that the DNA will not be degraded; hence, SLE is a defective DNA degradation disorder. Additionally, simultaneously high levels of anti-dsDNA, anti-nucleosome, and anti-histone antibodies in serum suggest that defective DNA degradation marks the genesis of SLE (19) and can indicate severe nephropathy in SLE. In summary, these findings improve our understanding of the role of T cells in SLE (20). During SLE pathogenesis, autoreactive T cells trigger the differentiation, proliferation, and maturation of B cells, thus supporting the formation of autoantibodies. Moreover, attenuating T cells normally alleviates the manifestations of autoimmune diseases, such as clearing pathogenic autoreactive T cells in SLE.

### Interferons and Defective DNA Degradation

The presence of pathogens, including lupus autoantigens, stimulates host cells to make and release a group of signaling proteins (cytokines) called IFNs through complex processes. There are three types of IFNs, namely, alpha, beta, and gamma IFNs. Gamma IFNs are known for the expansion of macrophages and inducing the class II major histocompatibility complex (MHC). Anti-IFN-γ autoantibodies are associated with amplified disease activity in patients with SLE (21). IFN-γ is commonly referred to as a type II IFN, whereas IFN-α and IFN-β are referred to as type I IFNs, a subtype of cytokines that help regulate the immune system.

Type I IFNs generated during a viral infection trigger the production of MCP-1, which is responsible for inducing the migration of inflammatory monocytes to the inflammation site. After recruitment, inflammatory monocytes are triggered by type I IFNs to generate IL-18, which then signals via IL-18R expressed by NK cells to induce the production of IFN-γ.

Abundant autoantibody production and IC formation cause tissue damage; hence, SLE is regarded as a B cell disorder. Conversely, the significance of helper T cells in inducing B cell immunity was identified based on defective CD3ζ function or expression. Reduced CD3ζ-chain expression has been identified in cancer patients and those with autoimmune illnesses. In contrast, decreased CD3ζ-chain levels are maintained during the course of SLE in most patients (22).

Moreover, IFN regulatory factor 5 (IRF5) plays a crucial role in the stimulation of type I IFNs, TNF-α, and the proinflammatory cytokines IL-6 and IL-12. It is also involved in adaptive and innate immunity. The first identified single-nucleotide polymorphism (SNP) in IRF5, Rs2004640, is closely related to the high expression of several IRF5 isoforms and is a valuable genetic risk factor for SLE (23).

Gene expression studies have identified hypomethylated upregulated genes and hypermethylated downregulated genes in SLE patients, indicating that DNA methylation plays a role in SLE development (24). Differentially expressed genes (differential gene co-expression) in SLE are involved in the IFN and TLR signaling pathways (24). The transcriptional repressor growth factor independence 1 (Gfi1) plays a critical role in myeloid cell regulation, as well as in the prevention of unprompted lupus autoimmunity, mainly by negatively regulating TLR7 signaling (58).

Before therapy with Gfi1, SLE mice showed high levels of serum autoantibodies specific for DNA and RNA, which are indicative of defective nucleic acid degradation in SLE. Hence, IFNs are involved in the etiopathogenesis of SLE by stimulating the formation of NETs, the main sources of cfDNA, and exploring their mechanisms of involvement has therapeutic potential (58).

### The Role of DNA Degradation in SLE

Suurmond et al. (59) maintained that studying genetic risk alleles can improve our understanding of failed tolerance mechanisms for DNA-reactive B cells and B cell alterations in SLE. Understanding the complexity of interactions between immune disturbance in SLE and epigenetic mechanisms is a potential way to explore new therapeutic targets (60). In particular, the digestion of chromatin in apoptotic cell microparticles (25) marks a tolerance mechanism for SLE. The absence of DNASE1L3, a DNA degrading enzyme, results in the production of anti-dsDNA antibodies specific for serum dsDNA; hence, defective DNA degradation is associated with SLE (25). When this process reaches a point at which autoantibodies are produced because of defective clearance of NETs and apoptotic remnants, the person is said to have global loss of self-tolerance (25). This is the point at which impaired DNA degradation plays a role in the etiopathogenesis of SLE.

Nuclear autoantigens (e.g., antigens specific for DNA and histones) (61) and proteins from the cytoplasm of neutrophils (62) play a role in the etiopathogenesis of SLE when cellular material containing these components leaks to the extracellular space through apoptosis or NETosis, hence exposing these factors to the immune system. Histones, which are a group of chromatin proteins that are abundant in NETs and apoptotic blebs, are modified through various processes, such as methylation (63), acetylation (64), ubiquitination (65), and poly-ADP ribosylation (66), during apoptosis, and NETosis. When modified histones are exposed to the immune system in the extracellular space, they are recognized as foreign and dangerous by receptors of the innate immune system, such as TLRs. Therefore, the H4/PK assay is usually a reliable and simple test that is valuable for the differential diagnosis and evaluation of symptomatic activity in SLE patients (26).

On the other hand, DNA methylation, an epigenetic mechanism for controlling gene expression (67), plays a significant role in the etiopathogenesis of SLE. Abnormal DNA methylation in immune-related cells is associated with SLE and can be detected in unfractionated white blood cells, which has diagnostic implications (27). This abnormal DNA methylation in immune-related cells involves altered DNA methylation of cytokine genes, which makes the chromatin inaccessible for destruction (60).

Moreover, SLE is associated with decreased DNA methyltransferase mRNA expression levels (18), which is the exact mechanism through which abnormal DNA methylation occurs in immune-related cells. Therefore, DNA hypomethylation of immune-related cells plays a key role in the etiopathogenesis of SLE; however, little is known about hypermethylation in SLE.

Only one study examined the role of CD3Z (a T cell surface glycoprotein and component of the T cell antigen receptor) hypermethylation in SLE, and it was determined that this modification is a potential risk factor associated with severe SLE manifestations (28).

Hypermethylation of CD3Z could be an environmentally induced epigenetic modification (22). In particular, CD3Z hypermethylation could provide an important mechanism for CD3ζ-chain downregulation in SLE T cells. Furthermore, CD3Z DNA hypermethylation was connected to more severe clinical signs in persons with SLE; thus, CD3Z hypermethylation is a marker of disease severity (22).

### Other Factors Related to Defective DNA Degradation

Many other factors have recently been associated with the etiopathogenesis, diagnosis, and treatment of SLE. There is extensive evidence for the association of IFNs and ILs with SLE; however, little is known about the role of TNFs (additional cytokines) in the etiopathogenesis of SLE. Variants of TNF ligand superfamily member 13B (TNFSF13B) are associated with defective DNA degradation in SLE disease development. Elevated serum levels of TNFSF13B correlate with serum dsDNA antigen levels, suggesting that TNF is associated with abnormal DNA degradation in SLE patients (28).

Moreover, recent studies have shown that some types of drugs can treat or contribute to the etiopathogenesis of SLE. Rituximab, which has proved efficacious in the treatment of diffuse alveolar hemorrhage in SLE, can cause IgM hypogammaglobulinemia, which can be predicted by lower baseline serum IgM levels and sequential therapy with mycophenolate mofetil (MMF) (29). This finding shows that high SLE disease activity is associated with dsDNA antigens in serum, suggesting defective DNA degradation. Early treatment with hydroxychloroquine can curb the development of endothelial dysfunction in SLE patients (30). Bortezomib, an anticancer drug, increases proinflammatory cytokine levels in mice, hence worsening SLE (31). Aconitine, a C19 norditerpenoid alkaloid, can inhibit disease progression and ameliorate pathologic lesions of SLE (32). This alkaloid, which is the key active component of *Aconitum*, has immunomodulatory properties and may be valuable for treating autoimmune diseases such as SLE (32).

TREX1 frame-shift mutations, such as V235fs and D272fs, trigger autoantibodies chiefly against non-nuclear antigens. Thus, patients who have such mutations are possibly ANA negative. High serum ANA levels are very rare among patients who have retinal vasculopathy with cerebral leukodystrophy (RVCL). Thus, diseases associated with such mutations may be under- or misdiagnosed. The oligosaccharyltransferase (OST) inhibitor aclacinomycin effectively suppressed the production of autoantibodies, confirming the possible therapeutic value of such medicines for treating phenotypes associated with the V235fs mutation. Moreover, the D272fs and V235fs mutations usually affect the DNase-independent roles of TREX1 and trigger serologic autoimmunity, which likely contributes to the development of autoimmune disease (33, 34).

In addition, IFN-α expression is positively related to IRF5 expression. Childhood-onset SLE normally follows a more aggressive course, with higher mortality and morbidity and a greater occurrence of serological and immunological abnormalities, than does adult-onset SLE, perhaps implicating dissimilar mechanisms in both clusters. Nonetheless, IRF5 plays an important role in childhood-onset SLE pathogenesis. Sp1 can increase IRF5 promoter activity and mRNA expression. Sp1 expression is increased in childhood-onset SLE and is positively related to IRF5 levels; thus, high Sp1 expression might contribute to high IRF5 levels, and the subsequent high IFN-α levels are a fundamental feature of SLE pathology (68).

### DNase Defects Have Been Correlated to Lupus Erythematosus

**DNase I**, an endonuclease, is usually secreted to cleave extracellular DNA to tetranucleotides with 3′ hydroxyl DNA ends and 5′ monophosphate (68). Moreover, during apoptosis, DNase I is the specific endonuclease that facilitates chromatin breakdown. Together with the plasminogen system, DNase I enables prompt and efficient chromatin breakdown through the instantaneous degradation of DNA and DNA-binding proteins (68).

DNase I mutations have been associated with a familiar form of SLE (69).

**DNase II** is vital for self-dsDNA clearance in lysosomes to circumvent an immune response. Moreover, DNase II knockout is usually embryonically lethal in mice due to uncontrolled inflammation, whereas cGAS deletion in DNase II-deficient mice salvages this lethal phenotype. DNASEII defects are associated with autoinflammtion, but however have so far not been linked to the classical lupus phenotype.

TREX1, also known as **DNase III**, is a DNA exonuclease that engages in cytoplasmic dsDNA and ssDNA clearance. Loss-of-function mutations of TREX1 result in the accumulation of self-DNA and autoimmune diseases, such as SLE. **Mutations in DNase III/TREX1** have also been associated with a familiar form of lupus erythematosus (70–72).

TREX1-deficient mice display markedly reduced survival due to unprompted inflammatory myocarditis, increased circulatory failure and cardiomyopathy Trex1 prevents the cell-intrinsic initiation of autoimmunity in the mice model of TREX1-deficient mice (73–76).

Depletion of **STING**, IRF3, cGAS, or IFN-I receptor in TREX1-deficient mice protects against autoimmune disorders and death, indicating that cGAS–STING–IRF3 axis-regulated IFN-I production is responsible for autoimmune disorder development in TREX1-deficient mice. Nevertheless, in STING-deficient lupus mice, serum cytokine levels, autoantibody production, and lymphoid hypertrophy increase significantly in comparison to STING-adequate littermates.

Mutations leading to STING activation cause autoinflammation and can lead to an inflammatory vascular and pulmonary syndrome (77) and has also been linked to a familiar form of lupus erythematosus (78, 79).

But on the other hand STING deficiency can also lead to autoimmunity mediated by enhanced TLR activation in lupus prone mice (80). In this case STING potently suppresses inflammation in a model of SLE. This controversial behavior points out the complexity of regulation and is important to be considered for the development of targeted therapies directed against STING.

ANAs are considered the serological characteristic of SLE. These antibodies often bind various nuclear antigens, including DNA, histones, non-histone proteins and protein complexes with RNA and DNA. Due to the frequent manifestation of ANA abnormalities in SLE, ANA analysis is a vital element of clinical assessments and determinations of suitability for clinical tests or the use of particular therapies (34).

DNase I, an endonuclease, is usually secreted to cleave extracellular DNA to tetranucleotides with 3′ hydroxyl DNA ends and 5′ monophosphate (68). Moreover, during apoptosis, DNase I is the specific endonuclease that facilitates chromatin breakdown. Together with the plasminogen system, DNase I enables prompt and efficient chromatin breakdown by the instantaneous degradation of DNA and DNA-binding proteins (68).

Self-dsDNA is involved in SLE pathogenesis; these molecules are normally cleared in apoptosis, necrosis and NETs, but these processes are defective in SLE patients, thereby triggering the production of autoantibodies through unknown mechanisms. In addition, anti-dsDNA antibodies are formed by autoreactive B cells and autoreactive T cells. dsDNA ICs and autoantibodies stimulate robust IFN-I production via several intercellular DNA sensors, primarily in PDCs and phagocytes (81).

## Conclusion

The etiopathogenesis of SLE is still controversial, and there are limited therapeutic inventions for this disease. However, this paper has demonstrated that SLE is a DNA degradation disorder. In normal apoptosis, DNA and other nucleic materials are efficiently cleared through degradation and other complex mechanisms, blocking the formation of nuclear autoantigens. Autoantibodies produced against cfDNA are responsible for tissue and organ damage in SLE patients.

## Data Availability

Publicly available datasets were analyzed in this study. This data can be found here: https://www.ncbi.nlm.nih.gov/pubmed/.

## Author Contributions

The author confirms being the sole contributor of this work and has approved it for publication.

### Conflict of Interest Statement

The author declares that the research was conducted in the absence of any commercial or financial relationships that could be construed as a potential conflict of interest.

## Abbreviations

ANAs: Antinuclear antibodies
ATG5: Autophagy related 5
BAF: Barrier-to-autointegration factor
CD3Z: Cluster of differentiation 3Z
CD40: Cluster of differentiation 40
CD40L: Cluster of differentiation 40 ligand (CD40L)
cfDNA: Circulating free DNA
DAMPs: Damage associated molecular patterns
DNA: Deoxyribonucleic acid
DNASE II: Deoxyribonuclease II
DNASE III: Deoxyribonuclease III
DNASE I: Deoxyribonuclease I
DNMT1: DNA methyltransferase 1
DNMT3B: DNA methyltransferase 3 beta
DUSP23: Dual-specificity protein phosphatase 23
Gfi1: Growth factor independence 1
ICF: Immunodeficiency-centromeric instability-facial anomalies
ICs: Immune complexes
IFN-γ: Interferon gamma
IRF5: Interferon regulatory factor 5
MHC: Major histocompatibility complex
MMF: Mycophenolate mofetil
mtDNA: Mitochondrial DNA
NETs: Neutrophil extracellular traps
NIC: NF-κB-inducing kinase
NK: Natural killer
PDCs: Plasmacytoid dendritic cells
RNA: Ribonucleic acid
RVCL: Retinal vasculopathy with cerebral leukodystrophy
SLE: Systemic lupus erythematosus
SNP: Single-nucleotide polymorphism
STAT: Signal transducer and activator of transcription
STING: Stimulator of interferon genes
TLR: Toll-like receptor
TNFRSF: Tumor necrosis factor receptor superfamily
TNF-α: Tumor necrosis factor alpha
TREX1: Three prime repair exonuclease 1.
